# Association of longitudinal risk profile trajectory clusters with adipose tissue depots measured by magnetic resonance imaging

**DOI:** 10.1038/s41598-019-53546-y

**Published:** 2019-11-18

**Authors:** Susanne Rospleszcz, Roberto Lorbeer, Corinna Storz, Christopher L. Schlett, Christa Meisinger, Barbara Thorand, Wolfgang Rathmann, Fabian Bamberg, Wolfgang Lieb, Annette Peters

**Affiliations:** 1 Institute of Epidemiology, Helmholtz Zentrum München, German Research Center for Environmental Health, Neuherberg, Germany; 2 Department of Radiology, Ludwig-Maximilians-University Hospital, Munich, Germany; 3 Department of Diagnostic and Interventional Radiology, University of Tuebingen, Tuebingen, Germany; 4 Department of Diagnostic and Interventional Radiology, Medical Center-University of Freiburg, Faculty of Medicine, University of Freiburg, Freiburg, Germany; 5 Chair of Epidemiology, Ludwig-Maximilians-University München, UNIKA-T Augsburg, Augsburg, Germany; 6 German Center for Diabetes Research (DZD), München-Neuherberg, Neuherberg, Germany; 7 Institute for Biometrics and Epidemiology, German Diabetes Center, Duesseldorf, Germany; 8 Institute of Epidemiology and Biobank PopGen, Kiel University, Kiel, Germany; 9 German Centre for Cardiovascular Research (DZHK e.V.), Munich, Germany; 10 Chair of Epidemiology, Ludwig-Maximilians-University München, Munich, Germany

**Keywords:** Diagnostic markers, Risk factors

## Abstract

The objective of the study was to identify associations of longitudinal trajectories of traditional cardiometabolic risk factors with abdominal and ectopic adipose tissue depots measured by magnetic resonance imaging (MRI). We measured total abdominal, visceral, and subcutaneous adipose tissue in liter and intrahepatic, intrapancreatic and renal sinus fat as fat fractions by MRI in 325 individuals free of cardiovascular disease at Exam 3 of a population-based cohort. We related these MRI measurements at Exam 3 to longitudinal risk profile trajectory clusters, based on risk factor measurements from Exam 3, Exam 2 (seven years prior to MRI) and Exam 1 (14 years prior to MRI). Based on the levels and longitudinal trajectories of several risk factors (blood pressure, lipid profile, anthropometric measurements, HbA1c), we identified three different trajectory clusters. These clusters displayed a graded association with all adipose tissue traits after adjustment for potential confounders (e.g. visceral adipose tissue: β_ClusterII_ = 1.30 l, 95%-CI:[0.84 l;1.75 l], β_ClusterIII_ = 3.32 l[2.74 l;3.90 l]; intrahepatic: Estimate_ClusterII_ = 1.54[1.27,1.86], Estimate_ClusterIII_ = 2.48[1.93,3.16]. Associations remained statistically significant after additional adjustment for the risk factor levels at Exam 1 or Exam 3, respectively. Trajectory clusters provided additional information in explaining variation in the different fat compartments beyond risk factor profiles obtained at individual exams. In conclusion, sustained high risk factor levels and unfavorable trajectories are associated with high levels of adipose tissue; however, the association with cardiometabolic risk factors varies substantially between different ectopic adipose tissues. Trajectory clusters, covering longitudinal risk profiles, provide additional information beyond single-point risk profiles. This emphasizes the need to incorporate longitudinal information in cardiometabolic risk estimation.

## Introduction

Obesity is an established risk factor for several disease conditions, including cardiovascular disease (CVD) and type 2 diabetes^1,2^, and clusters with other traditional CVD risk factors, such as hypertension and hypercholesterolemia^3^.

Easily applicable metrices such as body mass index (BMI) and waist circumference (WC) are often used as measures of obesity. However, both BMI and WC do not sufficiently reflect the distribution of fat in the body, nor can they adequately quantify the amount of metabolically active adipose tissue^4^. For an appropriate quantification of the amount and distribution of adipose tissue, non-invasive imaging is increasingly being used, including magnetic resonance imaging (MRI)^5,6^.

In addition to subcutaneous adipose tissue (SAT), also visceral adipose tissue (VAT) and ectopic fat depots, i.e. the accumulation of adipose tissue in and around organs, might have local as well as systemic metabolic effects and, thereby, modulate overall cardiometabolic risk^7–9^.

Prior studies have indeed shown that accurate quantification of adipose tissue, including VAT and SAT, provides additional value in the prediction of cardiometabolic outcomes (e.g. incident CVD, type 2 diabetes), beyond anthropometric measures^10–13^.

The relation of other ectopic fat depots with traditional markers of cardiometabolic risk is less established, but supported by more recent evidence. Hepatic fat has been reported to be cross-sectionally associated with hypertension^14^, dyslipidemia^15^ and impaired glucose homeostasis^16^. Pancreatic fat showed associations with general obesity and diabetes^17–19^. For renal sinus fat, associations with hypertension and increased triglycerides have been reported^20–22^.

However, these associations of ectopic fat depots with cardiometabolic risk factors were only obtained in cross-sectional analyses and with varying imaging methodologies. It is not well known how the longitudinal exposure to multiple CVD risk factors relates to established MRI-derived fat compartments such as VAT and SAT, or to ectopic adipose tissue depots such as renal sinus fat. Therefore, in the present manuscript, we aimed to analyze the association of traditional CVD risk factor trajectories over a 14-year time horizon with different measures of adipose tissue, as determined by MRI. These fat measures include total adipose tissue (TAT), VAT, SAT, but also rather novel markers of ectopic fat, including renal sinus fat fraction (RSFF), intrahepatic fat fraction (HFF) and intrapancreatic fat fraction (PFF).

The trajectories of multiple cardiometabolic risk factors over time can be used to identify distinct longitudinal patterns, i.e clusters, reflecting different cumulative cardiometabolic risk factor exposure.

In our analyses, we aim to identify distinct longitudinal risk profile trajectory clusters, quantify the association of these clusters with adipose tissue traits and to determine their incremental value compared to single-point measurements of individual risk factors.

## Methods

### Study sample

We used longitudinal data from the KORA (Cooperative Health Research in the Region of Augsburg) S4 sample, a population-based cohort from Bavaria, Germany. The cohort has been examined repeatedly at three different time points (Exam 1, Exam 2, Exam 3). The sampling scheme and the examination protocols of the KORA cohorts have been previously described in detail^23,24^. The baseline examination, Exam 1, was conducted in 1999–2001 and comprised 4261 participants; Exam 2 took place in 2006–2008 with 3080 participants; and Exam 3 was conducted in 2013–2014, including 2279 participants. At Exam 3, a whole-body MRI was obtained in a subsample of 400 participants free of CVD^5^. This MRI sub-sample included a high proportion of participants with prediabetes (26%) and diabetes (14%), because a specific aim of the KORA-MRI substudy was to evaluate subclinical CVD burden in individuals with prediabetes and diabetes^5^.

All study participants provided written informed consent. The study was approved by the ethics committee of the Bavarian Chamber of Physicians and the ethics committee of the Ludwig-Maximilians-University Munich and complies with the Declaration of Helsinki.

For the present analyses, a total of 75 of the 400 KORA-MRI study participants had to be excluded, because they did not participate in Exam 2 (n = 20) or because of missing values in any of the MRI parameters of interest (n = 55). Missing values in the MRI parameters were due to insufficient image quality, imaging artifacts and technical errors and were unrelated to the participants’ clinical covariates.

### Covariate assessment

At all three examinations, participants underwent a comprehensive physical examination, a blood draw and a standardized face-to-face interview conducted by trained examiners.

Height and weight were determined by Seca’s measuring systems (Seca GmbH & Co, KG, Hamburg, Germany) with either calibrated steelyards or digital scales. Height was quantified to the closest 0.1 cm and weight to the closest 0.1 kg. BMI was calculated as weight in kg divided by squared height in m.

WC was measured with an inelastic tape at the level midway between the lower rib margin and the iliac crest. Hip circumference was measured at the level of maximal gluteal protrusion.

Blood pressure was measured on the right upper arm using an OMRON type HEM-705CP oscillometric device. After at least 5 minutes of rest, three measurements were taken at intervals of three minutes. The mean of the second and third blood pressure measurement was used for the present analyses. Hypertension was defined as systolic/diastolic blood pressure above 140/90 mmHg or intake of antihypertensive medication, given that the participant was aware of being hypertensive. Antihypertensive medication was defined according to German national guidelines^25^.

Laboratory measurements have been described previously^26^. Briefly, for the assessment of total cholesterol, LDL cholesterol and HDL cholesterol, enzymatic, photometric assays were used at Exam 1, and enzymatic, colorimetric Flex assays were used at Exam 2 and Exam 3. HbA1c was measured by a turbidimetric inhibition immunoassay at Exam 1 and by cation-exchange high performance liquid chromatographic, photometric assays at Exam 2 and Exam 3. Supplementary Table S1 provides a more detailed description of the laboratory measurements.

Diabetes status, cigarette consumption, physical activity, alcohol intake and medication intake were self-reported. Participants were labeled as being physically active if they reported engaging in sports activities regularly for ≥1 hour per week or as physically inactive if they reported engaging in sports activities irregularly and less than 1 hour per week. At Exam 3, glycemic status was additionally validated by an oral glucose tolerance test and categorized into normoglycemic, prediabetes or diabetes according to the WHO guidelines^27^.

For our analysis, we used the following traits as traditional CVD risk factors: systolic blood pressure, diastolic blood pressure, BMI, WC, total cholesterol, HDL, LDL and HbA1c.

### MRI outcome assessment

The whole-body MRI protocol of the KORA-MRI substudy has been previously described in detail^5^. In brief, all MRI scans were performed on a 3 Tesla Magnetom Skyra (Siemens Healthineers, Erlangen, Germany) using a table-mounted spine matrix coil together with an 18-channel whole-body radiofrequency coil. The whole-body MRI protocol comprised multiple sequences to cover head, cardiovascular system and abdominal region. All images were read by independent radiologists blinded to the participants’ clinical covariates and standard quality measures of inter-and intrareader variability were evaluated.

**Figure 1:**
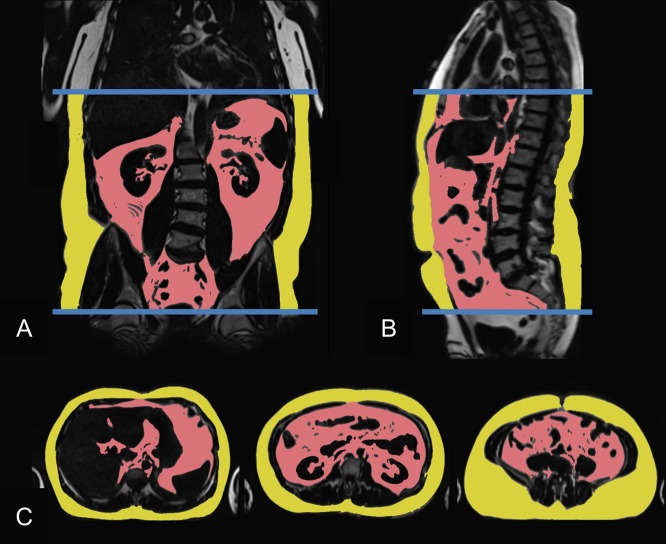
MRI-based assessment of visceral (VAT; red) and subcutaneous (SAT; yellow) adipose tissue in a 46-year-old male (VAT 6.57 l), displayed in coronar (**A**), sagittal (**B**) and axial (**C**) slices.

For quantification of adipose tissue compartments, volume-interpolated 3D in/opposed-phase VIBE-Dixon sequence was performed and adipose tissues were segmented semi-automatically^28,29^. SAT was quantified from cardiac apex to femoral head and VAT was quantified from diaphragm to femoral head; Total adipose tissue was defined as the sum of SAT and VAT, all indicated in liter. Figure 1 exemplifies the segmentation and quantification of VAT and SAT.

**Figure 2:**
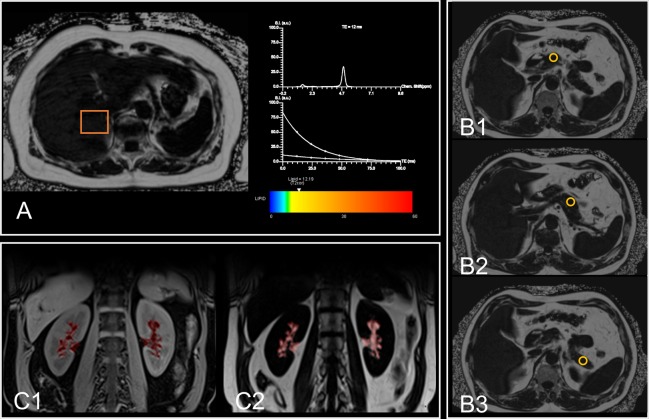
Exemplary MRI images of ectopic fat quantification. (**A**) Quantification of hepatic fat fraction. The dual-echo Dixon sequence shows the region of interest (orange square) placed in the liver parenchyma on the level of the portal vein. Results of the multi-echo spectroscopy are displayed as graph and colored bar. (**B**) Quantification of pancreatic fat fraction. Using a multi-echo Dixon-VIBE sequence, circular regions of interest were drawn into the pancreatic caput (B1), corpus (B2) cauda (B3) and resulting proton-density fat fractions were averaged. (**C**) Quantification of renal sinus fat fraction. Displayed is the overlay of renal sinus segmentation with Water-Only (C1) and Fat-Only (C2) Dixon images.

For the determination of HFF in %, a multi-echo Dixon-VIBE T1-weighted sequence was used, accounting for confounding T2* decay and spectral complexity of fat^14^. HFF was calculated as the mean fat fraction of right liver lobe (measured in segment VIII according to Couinaud classification) and left liver lobe (measured in segment II). An exemplary MRI image of HFF quantification is shown in Fig. 2A.

PFF was measured by the 3D multi-echo Dixon-VIBE sequence by drawing regions of interest into the pancreatic head, body and tail, and was measured as proton-density fat fraction in %^30^. An exemplary MRI image of PFF quantification is shown in Fig. 2B.

Additionally, based on the volume-interpolated 3D in/opposed-phase T1 weighted VIBE-Dixon sequence, an inhouse MATLAB algorithm was used for semi-automatic segmentation of total renal volume, renal cortex, medulla and sinus^31^. RSFF was then determined by overlaying the segmentation with the respective Water-Only and Fat-Only Dixon images. An exemplary MRI image is shown in Fig. 2C.

### Statistical analysis

#### Descriptive statistics

Continuous variables are summarized by arithmetic means and standard deviation (SD) and categorical variables are presented as counts and percentages. Differences in variables between Exam 1, Exam 2 and Exam 3 were evaluated by repeated measures ANOVA and Cochran’s Q-Test, respectively. MRI-derived adipose tissue outcome variables at Exam 3 are summarized as mean and SD or median with interquartile range.

We created models based on single-point risk profiles and models based on longitudinal trajectory clusters to be able to determine the additional value of longitudinal trajectories.

#### Association of single-point risk profiles at individual Exams with adipose tissue

To assess the association of single-point risk profiles at Exam 1, Exam 2 and Exam 3 (each Exam considered separately) with the different adipose tissue variables as outcomes, linear regression models were constructed. Due to their skewed distribution, HFF and PFF were log-transformed prior to analysis. The risk profile included eight variables: systolic blood pressure, diastolic blood pressure, BMI, WC, total cholesterol, HDL, LDL and HbA1c. The outcome variables comprised TAT, SAT, VAT, RSFF, log (HFF) and log (PFF). As adjustment covariates, the models included age, sex, antihypertensive medication, lipid-lowering medication, smoking behavior and diabetes. All continuous variables were standardized (mean = 0, sd = 1) prior to analysis. The Goodness-Of-Fit statistic R^2^ served as a measure of how much variance in the outcome is explained by the respective statistical model.

#### Identification of longitudinal risk factor clusters

We then identified longitudinal trajectories of risk factor profiles, using information from Exam 1, Exam 2 and Exam 3 simultaneously. Longitudinal risk factor trajectories were computed by unsupervised non-parametric k-means clustering using Euclidean distance^32^. Briefly, participants within one cluster should be similar to each other with regard to their risk profile, and dissimilar from participants in other clusters. We used Euclidean distance to determine the closeness of risk factor values between individuals and a mathematical norm function to determine the similarity of the resulting risk factor vectors^33^. Importantly, this automated clustering algorithm is unbiased and does not depend on pre-determined risk factor value cut-offs. Based on visual inspection and Calinski-Harabasz criterion, the optimal number of clusters was three. According to their individual multivariate trajectories, each study participant was automatically assigned to one of the three clusters, denoted by Roman Numericals as Cluster I, Cluster II and Cluster III.

#### Association of longitudinal risk profile trajectories with adipose tissue

Subsequently, associations of the trajectory clusters with adipose tissue outcomes were evaluated by linear regression models adjusted for age, sex, antihypertensive medication, lipid-lowering medication, smoking status and diabetes, all of them measured Exam 3. The trajectory clusters entered the linear model as a categorical variable with three levels. Cluster I served as the reference category and estimates were obtained for Cluster II and Cluster III. Furthermore, in order to assess whether longitudinal trajectory clusters provided additional information beyond the risk factor values obtained at the beginning (Exam 1) or end of the study (Exam 3), we created two additional models: The above model was additionally adjusted for the risk profile of Exam 1 or additionally adjusted for the risk profile at Exam 3, respectively. For all models, R^2^ was calculated as a measure of variance explained.

Additionally, as sensitivity analyses, all analyses were repeated with BMI and WC excluded from the risk profile variables and instead used as outcomes variables.

Two sided p-values < 0.05 were considered to indicate statistical significance. All computations were performed with Stata 14.1 (Stata Corporation, College Station, Texas, USA) and R 3.4.1 (R Core Team, Vienna, Austria).

## Results

### Trends in risk factor profiles

The cardiometabolic risk factor profiles at Exam 1, Exam 2 and Exam 3 are presented in Table 1. The sample comprised 59.4% men; mean age at baseline was 42.2 years. Over the course of 14 years, mean systolic and diastolic blood pressure declined significantly, while the percentage of individuals treated with antihypertensive medication increased significantly. Mean body weight, WC and BMI increased (Table 1). Mean HbA1c and prevalence of diabetes increased, whereas total cholesterol decreased. Alcohol consumption remained stable and more participants quit smoking and became physically active. A description of the different MRI-derived adipose tissue traits that served as outcome variables is provided in Table 2.

**Table 1:** Cardiometabolic risk profile of the study sample (N = 325) at Exam 1, Exam 2 and Exam 3 (time of MRI examination)

	Exam 1	Exam 2	Exam 3	p-value
	(1999–2001)	(2006–2008)	(2013–2014)	
Men	193 (59.4%)	193 (59.4%)	193 (59.4%)	
Age, years	42.2 ± 9.2	49.2 ± 9.2	56.2 ± 9.2	
Systolic BP, mmHg	126.5 ± 16.4	121.3 ± 16.5	121.1 ± 16.4	<0.01
Diastolic BP, mmHg	81.6 ± 10.5	76.4 ± 9.6	75.6 ± 10.1	<0.01
Hypertension, # of individuals	94 (28.9%)	80 (24.6%)	109 (33.5%)	<0.01
Antihypertensive Treatment, # of individuals	25 (7.7%)	43 (13.2%)	77 (23.7%)	<0.01
BMI, kg/m^2^	26.6 ± 3.8	27.3 ± 4.2	28.0 ± 4.7	<0.01
Weight, kg	78.8 ± 13.3	81.4 ± 14.8	82.9 ± 16.0	<0.01
Waist Circumference, cm	90.4 ± 11.5	93.6 ± 12.9	98.3 ± 13.8	<0.01
Hip Circumference, cm	104.4 ± 6.8	106.0 ± 7.7	106.6 ± 8.5	<0.01
Waist-To-Hip-Ratio	0.9 ± 0.1	0.9 ± 0.1	0.9 ± 0.1	<0.01
Total Cholesterol, mg/dL	223.8 ± 40.1	214.6 ± 36.7	218.4 ± 36.9	<0.01
LDL Cholesterol, mg/dL	134.0 ± 39.0	137.6 ± 32.8	140.4 ± 33.0	n.s
HDL Cholesterol, mg/dL	56.1 ± 17.2	53.6 ± 14.2	61.7 ± 18.1	<0.01
Ratio Total Cholesterol/HDL	4.4 ± 1.6	4.2 ± 1.2	3.8 ± 1.3	<0.01
Ratio LDL/HDL	2.7 ± 1.2	2.7 ± 1.0	2.5 ± 1.0	0.01
Lipid-lowering Medication, # of individuals	5 (1.5%)	20 (6.2%)	32 (9.8%)	<0.01
Diabetes mellitus, self-reported, # of individuals	3 (0.9%)	14 (4.3%)	27 (8.3%)	<0.01
HbA1c, %	5.5 ± 0.5	5.5 ± 0.5	5.6 ± 0.7	0.02
Antidiabetic Medication, # of individuals	3 (0.9%)	8 (2.5%)	24 (7.4%)	<0.01
Alcohol consumption, g/day	19.5 ± 25.3	17.9 ± 23.5	18.3 ± 22.2	n.s
Smoking, # of individuals				0.04
never-smoker	121 (37.2%)	121 (37.2%)	121 (37.2%)	
ex-smoker	116 (35.7%)	133 (40.9%)	140 (43.1%)	
smoker	88 (27.1%)	71 (21.8%)	64 (19.7%)	
Physically active, # of individuals	161 (49.5%)	192 (59.1%)	198 (60.9%)	<0.01

Continuous variables are presented as mean and standard deviation with p-values calculated by repeated measures ANOVA, indicating whether the mean values differ significantly in at least two time points. Categorical variables are presented as counts and percentages with p-values calculated by Cochrans Q Test, indicating whether the percentage of subjects differ significantly in at least two time points.

**Table 2:** MRI-derived adipose tissue measures of the study sample (N = 325), obtained at Exam 3.

	N = 325
TAT, l	12.6 ± 5.3
VAT, l	4.5 ± 2.7
SAT, l	8.1 ± 3.6
RSFF, %	63.9 ± 9.9
HFF, % (median[IQR])	5.7 [3.0, 11.7]
PFF, % (median[IQR])	5.4 [3.4, 9.2]

TAT: Total adipose tissue, VAT: Visceral adipose tissue, SAT: Subcutaneous adipose tissue, RSFF: Renal sinus fat fraction, HFF: Hepatic fat fraction, PFF: Pancreatic fat fraction.

### Associations of single-point risk profiles with adipose tissue traits

**Figure 3:**
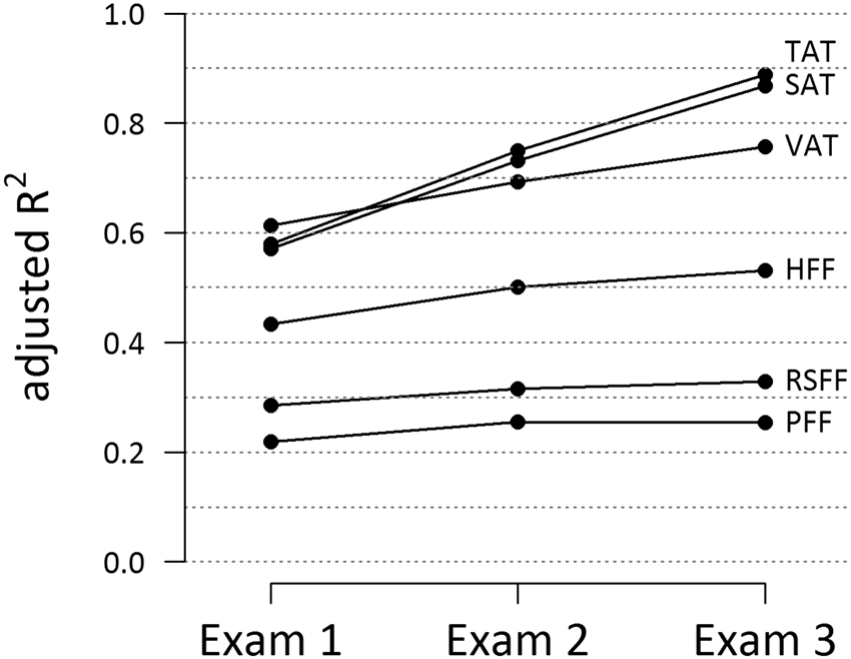
Goodness-of-Fit of the linear regression models estimating the association of single-point risk profiles with adipose tissue outcomes. On the x-axis: single time points at which risk profiles were obtained: Exam 1, Exam 2, Exam 3. On the y-axis: Goodness-of-Fit as measured by explained variance in outcome (adjusted R^2^). The single time points are connected by lines for visual aid only. The risk factor profiles included systolic blood pressure, diastolic blood pressure, BMI, WC, Total Cholesterol, HDL, LDL and HbA1c whereas the outcome variables comprised TAT, SAT, VAT, RSFF, log (HFF) and log (PFF).TAT: Total adipose tissue, VAT: Visceral adipose tissue, SAT: Subcutaneous adipose tissue, RSFF: Renal sinus fat fraction, HFF: Hepatic fat fraction, PFF: Pancreatic fat fraction

Figure 3 shows the ability of individual single-point risk profiles at Exam 1, Exam 2 and Exam 3, respectively, to explain the variance in the different adipose tissue traits. At each Exam, the risk profile explained only a modest proportion of the variation in pancreatic and renal sinus fat and the amount of variation explained by the risk factors was similar across Exams 1 to 3 (about 20% to 30% of variance explained, Fig. 3). For hepatic fat (HFF), a moderate proportion of variance was explained by the individual risk profiles with a slight increase from Exam 1 (above 40%) to Exam 3 (above 50%). For TAT, SAT and VAT, the individual risk profiles explained a large proportion of the variance in the adipose traits with a substantial increase from Exam 1 (around 60% of variance explained) to Exam 3 (almost 90% of variance explained for TAT).

In sensitivity analyses, when we excluded BMI and WC from the risk factor profiles, the amount of variance explained was considerably lower for all adipose tissue traits (compare Supplementary Figure S1). The highest R^2^ values were obtained for VAT, WC and HFF (all R^2^ > 0.4), whereas the values for SAT and BMI were substantially decreased.

### Characterization of longitudinal risk profile trajectory clusters

**Figure 4:**
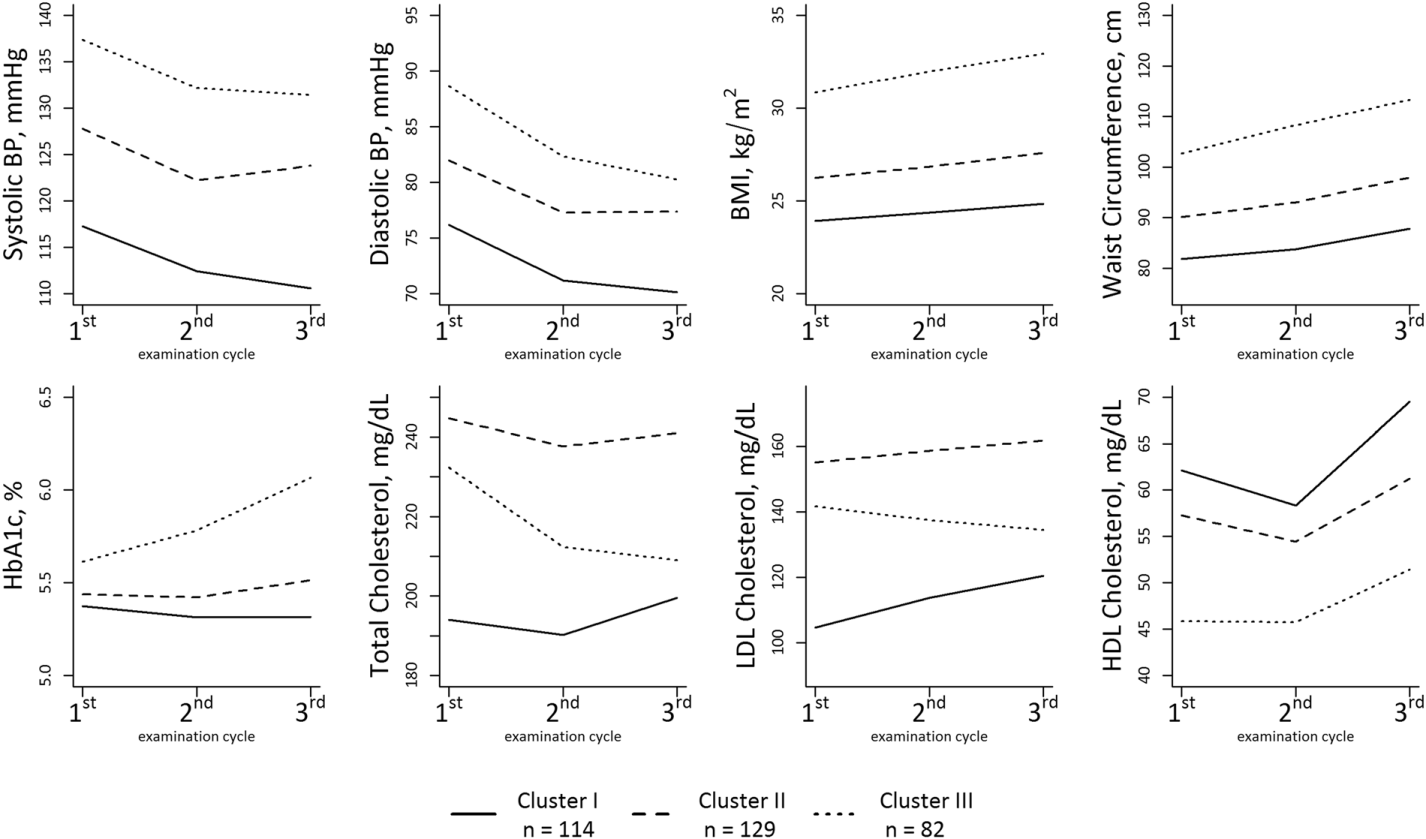
Mean risk factor levels at Exam 1, Exam 2 and Exam 3 according to cluster membership of participants. Cluster membership in either Cluster I, Cluster II or Cluster III was determined by multivariate k-means clustering based on individual longitudinal risk profile trajectories.

By multivariate longitudinal k-means clustering, three distinct clusters of risk profile trajectories over a time period of 14 years were identified. In essence, the clusters differ in mean risk factor levels and in change over time of the individual risk factors (Fig. 4 and Supplementary Table S2). Specifically, Cluster I comprises 114 individuals (35% of the overall sample) and represents the lowest cardiometabolic risk burden. It includes individuals with the youngest average age, and the lowest mean levels of systolic and diastolic blood pressure, WC, BMI, HbA1c, total and LDL cholesterol at baseline. In addition, Cluster I is characterized by the lowest increase of WC and BMI and the highest increase of lipid parameters over time. Cluster II comprises 129 individuals (40% of the overall sample). Mean age, mean blood pressure values, mean BMI and WC, HbA1c and HDL reside between Cluster I and Cluster III. However, total cholesterol and LDL values are higher in this cluster than in the other two clusters.

Cluster III, comprising 82 individuals (25% of the overall sample), has the highest mean age and highest levels of blood pressure, BMI, and WC as well as the lowest levels of HDL at Exam 1. Furthermore, BMI, WC and HbA1c increased over time with the highest % change of all clusters (see Supplementary Table S2).

### Association of longitudinal risk profile trajectory clusters to adipose tissue traits

**Figure 5:**
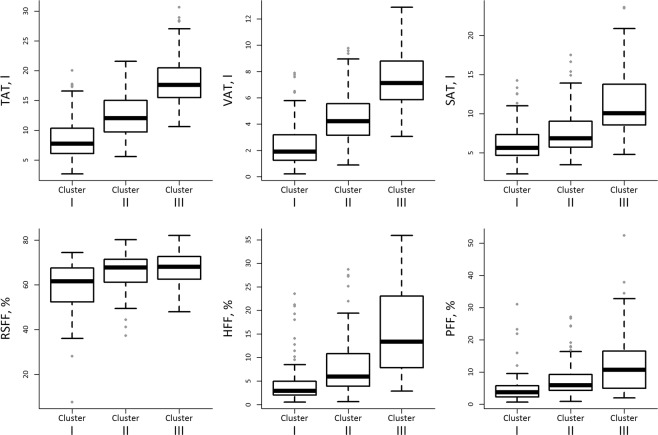
Box plots illustrating the distribution of adipose tissue depots, measured at Exam 3, according to cluster membership of participants. Cluster membership in either Cluster I, Cluster II or Cluster III was determined by multivariate k-means clustering based on individual longitudinal risk profile trajectories. TAT: Total adipose tissue, VAT: Visceral adipose tissue, SAT: Subcutaneous adipose tissue, RSFF: Renal sinus fat fraction, HFF: Hepatic fat fraction, PFF: Pancreatic fat fraction.

Figure 5 shows the distribution of the MRI-derived adipose tissue traits, measured at Exam 3, according to the three longitudinal trajectory clusters. Cluster I reflects low, Cluster II reflects moderate and Cluster III reflects high cumulative exposure to cardiometabolic risk factors over 14 years. A gradual increase in adipose tissue content for all traits is discernible from Cluster I to III, with the differences being statistically significant (all p < 0.001).

When we excluded BMI and WC from the risk factor set, the graded relation between the clusters with respect to the adiposity traits was less consistent (compare Supplementary Figure S2). Adipose tissue levels in Cluster I were lower compared to the other two clusters, but the average levels of different adipose tissue traits between Cluster II and III were comparable, e.g for TAT and SAT. Average BMI levels were similar between Cluster II and III (28.8 ± 4.2 kg/m^2^ and 29.4 ± 4.6 kg/m^2^, respectively), whereas WC levels were significantly lower in Cluster II compared to Cluster III (101.3 ± 11.4 cm vs 105.3 ± 13.1 cm, p = 0.02).

Upon multivariable adjustment for age, sex, antihypertensive and lipid-lowering medication, smoking and diabetes, the trajectory clusters were significantly associated with all adipose tissue outcomes (“fully adjusted” model in Table 3). For example, Cluster II was associated with an increase of 1.30 l in VAT (95%-CI 0.84 to 1.75) and a 2.60 l increase in SAT (95%-CI 1.87 to 3.34) whereas Cluster III was associated with an increase of 3.32 l in VAT (95%-CI 2.74 to 3.90) and a 6.16 l increase in SAT (95%-CI 5.23 to 7.10); always compared to Cluster I (Referent). In the same vein, Cluster II was associated with a 52% increase in PFF (95%-CI 26 to 84) and Cluster III was associated with a 120% increase in PFF (95%-CI 73 to 180), compared to Cluster I.

**Table 3:** Association of longitudinal risk profile trajectory clusters with different adipose tissue traits and impact of additional adjustment for risk factors obtained at Exam 1 or Exam 3.

outcome	Model	Cluster I	Cluster II			Cluster III			
			estimate	95%-CI	p-value	estimate	95%-CI	p-value	
TAT	fully adjusted	Ref.	3.90	[2.89, 4.91]	<0.01	9.49	[8.20, 10.78]	<0.01	R^2^ = 0.54
	fully adjusted + risk profile Exam 1	Ref.	3.21	[2.14, 4.29]	<0.01	6.25	[4.73, 7.77]	<0.01	R^2^ = 0.65
	fully adjusted+risk profile Exam 3	Ref.	0.39	[−0.26, 1.04]	n.s	0.96	[0.09, 1.82]	0.03	R^2^ = 0.89
VAT	fully adjusted	Ref.	1.30	[0.84, 1.75]	<0.01	3.32	[2.74, 3.90]	<0.01	R^2^ = 0.63
	fully adjusted + risk profile Exam 1	Ref.	1.16	[0.63, 1.69]	<0.01	2.62	[1.87, 3.37]	<0.01	R^2^ = 0.66
	fully adjusted + risk profile Exam 3	Ref.	0.17	[−0.30, 0.65]	n.s	0.90	[0.26, 1.53]	<0.01	R^2^ = 0.76
SAT	fully adjusted	Ref.	2.60	[1.87, 3.34]	<0.01	6.16	[5.23, 7.10]	<0.01	R^2^ = 0.48
	fully adjusted + risk profile Exam 1	Ref.	2.05	[1.30, 2.81]	<0.01	3.63	[2.56, 4.70]	<0.01	R^2^ = 0.63
	fully adjusted + risk profile Exam 3	Ref.	0.22	[−0.26, 0.70]	n.s	0.06	[−0.59, 0.71]	n.s	R^2^ = 0.87
RSFF	fully adjusted	Ref.	3.39	[1.02, 5.76]	<0.01	3.20	[0.18, 6.22]	0.04	R^2^ = 0.29
	fully adjusted + risk profile Exam 1	Ref.	2.47	[−0.41, 5.35]	n.s	0.31	[−3.77, 4.39]	n.s	R^2^ = 0.29
	fully adjusted + risk profile Exam 3	Ref.	0.19	[−2.80, 3.18]	n.s	−2.28	[−6.28, 1.72]	n.s	R^2^ = 0.33
HFF	fully adjusted	Ref.	1.54	[1.27, 1.86]	<0.01	2.48	[1.93, 3.16]	<0.01	R^2^ = 0.43
	fully adjusted + risk profile Exam 1	Ref.	1.51	[1.21, 1.90]	<0.01	2.23	[1.62, 3.03]	<0.01	R^2^ = 0.47
	fully adjusted + risk profile Exam 3	Ref.	1.14	[0.91, 1.43]	n.s	1.32	[0.98, 1.79]	n.s	R^2^ = 0.53
PFF	fully adjusted	Ref.	1.52	[1.26, 1.84]	<0.01	2.20	[1.73, 2.80]	<0.01	R^2^ = 0.24
	fully adjusted + risk profile Exam 1	Ref.	1.52	[1.21, 1.92]	<0.01	1.82	[1.32, 2.51]	<0.01	R^2^ = 0.26
	fully adjusted + risk profile Exam 3	Ref.	1.40	[1.10, 1.79]	<0.01	1.58	[1.15, 2.20]	<0.01	R^2^ = 0.27

Estimates are derived from a linear regression model. Cluster I served as reference category, i.e. estimates describe the change in adipose tissue outcome that is associated with membership in Cluster II (or Cluster III) as compared to membership in Cluster I. Estimates for TAT, VAT, SAT and RSFF are given as β-coefficients. Estimates for HFF and PFF are back-transformed from log-transformation and are therefore given as %change of the geometric mean. The fully adjusted model is adjusted for age, sex, antihypertensive medication, lipid-lowering medication, smoking status, and validated diabetes.

When BMI and WC were excluded from the risk factor set, associations were attenuated and less graded, e.g. Cluster II was associated with an increase of 1.09 l in VAT (95%-CI:[0.53; 1.66]) and a 1.57 l increase in SAT (95%-CI:[0.62; 2.53]) whereas Cluster III was associated with an increase of 1.09 l in VAT (95%-CI:[0.48; 1.70]) and a 1.71 l increase in SAT (95%-CI:[0.68, 2.74]).

After adjustment for the risk profile obtained at Exam 1, the associations of the clusters with adipose tissue traits were attenuated but remained highly statistically significant (Table 3). The model including both the trajectory cluster and the risk profile from Exam 1 explained more variation of the different fat compartments than the risk profile from Exam 1 alone (Supplementary Table S3). This indicates that the longitudinal information comprised in the clusters provides additional information, beyond the risk factor values measured at the beginning of the study (Exam 1).

After adjustment for the risk profile at Exam 3, trajectory clusters were still significantly associated with TAT, VAT and PFF (Table 3). Importantly, R^2^ measures for TAT, VAT, RSFF, HFF and PFF were higher compared to the model using solely the risk profile at Exam 3 (compare Supplementary Table S3). This indicates that the longitudinal information comprised in the clusters provides additional information, beyond the risk factor values measured concurrent to the MRI examination (Exam 3).

## Discussion

We analyzed longitudinal trajectories of multiple cardiometabolic risk factors by identifying multivariate clusters, and evaluated the association of these longitudinal risk profile trajectory clusters with a broad panel of MRI-derived abdominal and ectopic adipose tissue traits. Our main findings are threefold: First, a high and sustained cumulative risk factor exposure is associated with larger amounts of adipose tissue. Second, the variability in adipose tissue that is explained by traditional CVD risk factors varies substantially, with rather modest values for localized ectopic tissue depots such as renal sinus fat and pancreatic fat and high values for systemic metabolic organs such as VAT and SAT. Third, longitudinal risk profile information provides additional value, beyond single-point analyses.

### Identification of trajectory clusters

A clustering of CVD risk biomarkers is common^34,35^. However, data on the dynamic changes of several cardiometabolic risk factors over time are limited. We used an unbiased method, not depending on arbitrary cut-points, to classify individuals according to their longitudinal risk profile trajectories results by using a multivariate, unsupervised algorithm. We identified three clusters, which reflect low, medium and high cumulative risk factor exposure over 14 years. The identified trajectories are based on the mean risk factor values and the change in mean risk factor levels from Exam 1, Exam 2 and Exam 3.

### Association of longitudinal risk factor trajectories with MRI adipose tissue traits

Traditional CVD risk factors explained substantial amounts of variability in SAT, VAT and hepatic fat (all R^2^ > 50%, Fig. 3). These adipose tissue depots are highly metabolically active organs^36,37^. For smaller, more localized fat depots (renal sinus fat, pancreatic fat), longitudinal clusters of traditional CVD risk factors were strongly statistically associated, but could explain only a smaller amount of variability. This indicates different biological mechanisms and pathways in the relation of traditional CVD risk factors to these fat compartments.

#### Total abdominal, visceral and subcutaneous fat

In our study, both VAT and SAT were strongly associated with traditional CVD risk profiles. These observations are in line with results from the Jackson Heart Study, where SAT and VAT were cross-sectionally associated with hypertension, fasting plasma glucose, triglycerides and HDL^38,39^ and also with results from the Framingham Heart Study, where VAT and SAT were associated with hypertension, plasma glucose and lipid profile^22,40,41^. Our results corroborate that increased SAT is not associated with a more favorable cardiometabolic risk profile^39,42^. In our analysis, SAT had the highest R^2^ value of all adipose tissue depots, i.e. the highest amount of variation that could be explained by the risk profile. However, when BMI and WC were excluded from the risk profile, the R^2^ value for SAT became substantially smaller and was lower than that of VAT. The high value for SAT might therefore have been mainly driven by the high correlation of SAT with anthropometric measurements. This in line with other studies reporting an attenuated association of SAT to cardiometabolic risk markers after adjustment for BMI or WC^39,42,43^.

VAT contributes to CVD risk e.g. by elevated lipolytic activity, increased low-grade inflammation, and raised production of cytokines and other chemical messenger compounds^44^. Unfavorable health effects of excess abdominal adipose tissue are well established^45^. An important observation from our study is that longitudinal trajectory clusters were significantly associated with VAT, even in addition to the risk profile concurrent to the VAT quantification. Thus, our results highlight the importance of taking an individual’s risk profile history into account to more accurately quantify the cardiometabolic risk associated with VAT.

#### Hepatic fat fraction

In our study, more than half of the variance in hepatic fat could be explained by traditional CVD risk factors. In line with our observations, unfavorable BMI trajectories over the life course (25 years) were associated with an increased risk of developing non-alcoholic fatty liver disease (NAFLD) in the CARDIA study^46^. A correlation of NAFLD with general obesity is now well established^37^. A recent Chinese study found cross-sectional associations of NAFLD with overall obesity, increased lipids, impaired glucose homeostasis and uric acid^47^. The Framingham Heart Study reports associations of fatty liver with unfavorable glycemic traits and unfavorable lipid profile^15^. Moreover, a modulation of hepatic fat by VAT has been proposed^48,49^.

Formation of hepatic fat is a complex, long-term process with a multifactorial etiology^50^. Elevated hepatic fat is related to CVD, in part by its contribution to insulin resistance, increased free fatty acids and chronic inflammation^48,51^. An important finding from our study is that longitudinal trajectory clusters provided additional explanatory value for hepatic fat, beyond the baseline measurements and also beyond the measurements concurrent to the fat quantification, although this additional contribution was minor. Hence, our results underline the importance of accounting for an individual’s longitudinal risk factor levels to improve estimation of cardiometabolic risk associated with hepatic fat.

#### Pancreatic fat fraction

In our study, longitudinal risk profile trajectory clusters explained only a quarter of the variance in pancreatic fat, which was the lowest value of all adipose tissue depots analyzed. The interrelation of pancreatic fat and cardiometabolic disease conditions is not entirely clear^48,52^. For example, studies on the association of pancreatic fat with impaired glucose metabolism and type 2 diabetes are inconclusive^30,53^. Other studies have reported that pancreatic fat content is associated with serum triglyceride and nutritional fat intake^54^ and responsive to exercise and nutrition changes^55^. A recent meta-analysis of pancreatic steatosis found significant associations with metabolic syndrome, central obesity and hypertension, however with substantial heterogeneity between the studies^56^. Interestingly, although hepatic and pancreatic fat have been reported to be highly correlated^19,54^, we observed differential associations of liver and pancreatic fat with a traditional CVD risk profile.

Importantly, our study showed that longitudinal trajectory clusters were significantly associated with pancreatic fat, even in addition to the risk profile concurrent to pancreatic fat quantification.

Our results therefore emphasize that longitudinal risk factor information should be taken into account while further elucidating the role of this localized ectopic fat depot in the metabolism of traditional CVD risk factors.

#### Renal sinus fat fraction

We observed in our sample that approximately a third of the variance in renal sinus fat could be explained by traditional CVD risk factors. Very few studies have reported associations of traditional CVD risk factors with renal sinus fat. A clinical study including 51 NAFLD patients reported no significant association of renal sinus fat fraction to BMI, cholesterol, HDL and fasting glucose^57^. Similarly, in 205 participants of the PREDICT study, an association of renal sinus fat with the number of antihypertensive medications taken was reported, but no significant association to BMI or diabetes was observed^20^. In the Framingham Heart Study, associations of renal sinus fat as determined by Computed Tomography with hypertension, chronic kidney disease and increased triglyceride levels were reported^21,22^.

Adipose tissue in the renal sinus can lead to increased pressure on the renal vasculature and thereby to structural damage in the kidney^58^. Associations of renal sinus fat with blood pressure and renal function have been proposed^21,59^. However, the metabolic role of renal sinus fat has not been fully clarified. A recent clinical trial reported that ectopic fat depots were affected by overall weight loss, but renal sinus fat was not modified by specific interventions such as diet and physical activity^59^.

In our analysis, longitudinal risk factor trajectories provided no distinct additional explanatory value for renal sinus fat. Our results therefore emphasize that the long-term effects and development of renal sinus fat have to be further elucidated to understand the role of this localized ectopic fat depot in the metabolism of traditional CVD risk factors.

### Strengths and limitations

Limitations of our study include the relatively small sample size which did not allow subgroup analyses, e.g. sex-stratified analyses. Furthermore, we only included CVD risk factors that were available for all participants at all examination time points. We acknowledge that several other risk factors, such as triglycerides, glucose levels, inflammation markers or liver enzymes have been hypothesized to be associated with ectopic fat depots^42,43,47^. Besides, over the course of 14 years and three examination time points, laboratory methods, instruments and assays inevitably changed. This might have affected the values of our CVD risk factor measurements and thus the derived trajectories. For example, HDL values at Exam 3 are rather high, whereas values at Exam 2 are comparable to those of other German cohorts from the same time period^60–62^. Furthermore, MRI measurements were only available at Exam 3; therefore we could not assess the change in the different fat depots over time. It is likely that those individuals with unfavorable risk factor profiles also had the highest amounts of ectopic adipose tissue at baseline, but this cannot be examined given the available data. Analysis of longitudinal MRI measures of ectopic fat would provide higher-level evidence regarding the association with traditional CVD risk factors. However, longitudinal, whole-body MRI measurements from population-based cohort studies are still scarce^63^.

A major strength of our analyses is the longitudinal study design with repeated standardized assessment of several established CVD risk factors over a long time period and the availability of a broad panel of MRI-determined adipose tissue measures. Furthermore, MRI is considered to be the gold standard for accurate quantification of adipose tissue. Another major strength of our study is the use of an automated clustering algorithm which characterizes participants based on their cardiometabolic risk factor profiles, thus obtaining an unbiased classification.

### Translational potential and conclusions

In conclusion, we report three main findings with translation potential. First, unfavorable risk factor trajectories, representing long cumulative exposure to elevated levels of cardiometabolic risk factors, are associated with increased adipose tissue depots. This underscores the need for rigorous management of traditional CVD risk factors. Second, CVD risk factors explained less variability in localized fat depots compared to highly systemically active fat depots. This implicates different pathophysiological pathways of these fat depots in CVD risk factor metabolism and cardiometabolic risk. Third, longitudinal risk factor trajectories add incremental information, above and beyond risk profiles obtained at individual time points, regarding their association with adipose tissue depots. This emphasizes the need to incorporate an individual’s longitudinal risk factor information to obtain improved estimation of the cardiometabolic risk that is associated with these adipose tissue depots.

## Supplementary information

Supplementary Material to Association of longitudinal risk profile trajectory clusters with adipose tissue depots measured by magnetic resonance imaging

## Data Availability

The informed consent given by KORA study participants does not cover data posting in public databases. However, data are available upon request from KORA-gen (http://epi.helmholtz-muenchen.de/kora-gen/) by means of a project agreement. Requests should be sent to kora.passt@helmholtz-muenchen.de and are subject to approval by the KORA Board.
