# Supplementary material for: Association of longitudinal risk profile trajectory clusters with adipose tissue depots measured by magnetic resonance imaging
Source: Sci Rep. 2019 Nov 18;9:16972. doi: 10.1038/s41598-019-53546-y (PMC6861315; doi:10.1038/s41598-019-53546-y)

Supplementary Material to

Association of longitudinal risk profile trajectory clusters with adipose tissue depots measured by magnetic resonance imaging

Susanne Rospleszcz*[1], Roberto Lorbeer*[2,9], Corinna Storz[3], Christopher L. Schlett[4], Christa Meisinger[1,5], Barbara Thorand[1], Wolfgang Rathmann[6,7], Fabian Bamberg[2,4], Wolfgang Lieb‡[8], Annette Peters‡[1,9,10]

* These authors contributed equally

‡ These authors contributed equally

[1]Institute of Epidemiology, Helmholtz Zentrum München, German Research Center for Environmental Health, Neuherberg, Germany
[2]Department of Radiology, Ludwig-Maximilians-University Hospital, Munich, Germany
[3]Department of Diagnostic and Interventional Radiology University of Tuebingen, Germany
[4]Department of Diagnostic and Interventional Radiology, Medical Center-University of Freiburg, Faculty of Medicine, University of Freiburg, Freiburg, Germany
[5]Chair of Epidemiology, Ludwig-Maximilians-University München, UNIKA-T Augsburg, Augsburg, Germany
[6]German Center for Diabetes Research (DZD), München-Neuherberg, Germany
[7]Institute for Biometrics and Epidemiology, German Diabetes Center, Duesseldorf, Germany
[8]Institute of Epidemiology and Biobank PopGen, Kiel University, Kiel, Germany
[9]German Centre for Cardiovascular Research (DZHK e.V.), Munich, Germany
[10]Chair of Epidemiology, Ludwig-Maximilians-University München, Munich, Germany

Supplementary Table S1: Description of laboratory measurements at Exam 1, Exam 2 and Exam 3

		**Exam 1 (1999-2001)**	**Exam 2 (2006-2008)**	**Exam 3 (2013-2014)**
**Total Cholesterol**	Instrument	Hitachi 717	Dimension RxL	Dimension Vista 1500<br>Cobas c701/702
	Assay	CHOL	CHOL Flex	CHOL Flex<br>CHOL2
	Assay type	Enzymatic, photometric	Enzymatic, colorimetric	Enzymatic, colorimetric
	Manufacturer	Roche Diagnostics GmbH, Mannheim, Germany	Dade Behring Inc., Newark, USA	Siemens Healthcare Diagnostics Inc., Newark, USA<br>Roche Diagnostics GmbH, Mannheim, Germany
	Remarks			During the study period, laboratory instruments and assays were changed from Siemens to Roche. 122 samples were measured with both instruments. These were used to derive calibration formulas based on Passing-Bablok regression to calibrate the Siemens values to the Roche values.<br>For Total Cholesterol: Total Cholesterol [Roche] = 3.00 mg/dl + Total Cholesterol [Siemens] * 1.00 mg/dL
**HDL Cholesterol**	Instrument	Hitachi 717	Dimension RxL	Dimension Vista 1500<br>Cobas c701/702
	Assay	CHOL	AHDL Flex	HDLC Flex<br>HDLC3
	Assay type	Enzymatic, photometric	Enzymatic, colorimetric	Enzymatic, colorimetric
	Manufacturer	Roche Diagnostics GmbH, Mannheim, Germany	Dade Behring Inc., Newark, USA	Siemens Healthcare Diagnostics Inc., Newark, USA<br>Roche Diagnostics GmbH, Mannheim, Germany
	Remarks	Determination of HDL Cholesterol after precipitation of non-HDL Cholesterol by HDL-C reagents		During the study period, laboratory instruments and assays were changed from Siemens to Roche. 122 samples were measured with both instruments. These were used to derive calibration formulas based on Passing-Bablok regression to calibrate the Siemens values to the Roche values.<br>For HDL Cholesterol: HDL Cholesterol [Roche] = 2.40 mg/dl + HDL Cholesterol [Siemens] * 1.12 mg/dL
**LDL Cholesterol**	Instrument	Hitachi 717	Dimension RxL	Dimension Vista 1500<br>Cobas c701/702
	Assay	CHOL	ALDL Flex	LDLC Flex<br>LDL_C
	Assay type	Enzymatic, photometric	Enzymatic, colorimetric	Enzymatic, colorimetric
	Manufacturer	Roche Diagnostics GmbH, Mannheim, Germany	Dade Behring Inc., Newark, USA	Siemens Healthcare Diagnostics Inc., Newark, USA<br>Roche Diagnostics GmbH, Mannheim, Germany

	Remarks	Determination of non-LDL Cholesterol after precipitation of LDL Cholesterol by QUANTOLIP reagents (Immuno AG, Vienna, Austria). LDL Cholesterol is then calculated as the difference between Total and non-LDL Cholesterol.		During the study period, laboratory instruments and assays were changed from Siemens to Roche. 122 samples were measured with both instruments. These were used to derive calibration formulas based on Passing-Bablok regression to calibrate the Siemens values to the Roche values.<br>For LDL Cholesterol: LDL Cholesterol [Roche] = antilog (-0.13328 + log (LDL Cholesterol [Siemens] * 1.03051))
**HbA1c**	Instrument	Hitachi 717	Adams HA 8160 Hemoglobin Analysis System	VARIANT II TURBO Hemoglobin Testing System
	Assay	Tina-Quant HBA1C II	Cation-exchange high performance liquid chromatographic, photometric assay	VARIANT II TURBO HbA1c Kit - 2.0
	Assay type	Turbidimetric inhibition immunoassay		Cation-exchange high performance liquid chromatographic, photometric
	Manufacturer	Roche Diagnostics GmbH, Mannheim, Germany	Arkray Inc., distributed by A. Menarini Diagnostics, Florence, Italy	Bio-Rad Laboratories Inc., Hercules, USA
**Blood pressure**	Instrument	HEM-705CP	HEM-705CP	HEM-705CP
	Instrument type	Automatic	Digital	Digital
	Manufacturer	OMRON HEALTHCARE GmbH	OMRON HEALTHCARE GmbH	OMRON HEALTHCARE GmbH

Supplementary Table S2: Risk factor values at Exam 1, Exam 2 and Exam 3 in the three longitudinal risk profile trajectory clusters.

	Exam cycle	Cluster I<br>N = 114	Cluster II<br>N = 129	Cluster III<br>N = 82	p-value
%male	Exam 3	48 (42.1%)	82 (63.6%)	63 (76.8%)	< 0.01
Age, years	Exam 3	51.6 ± 7.9	58.4 ± 8.9	59.2 ± 8.9	< 0.01
Systolic BP, mmHg	Exam 1	117.3 ± 13.8	127.8 ± 12.1	137.4 ± 18.4	< 0.01
	Exam 2	112.4 ± 14.1	122.2 ± 14.3	132.2 ± 16.0	< 0.01
	Exam 3	110.6 ± 12.8	123.8 ± 14.6	131.4 ± 15.6	< 0.01
	Δ%	-5.6 [-11.7, 1.1]	-3.2 [-10.5, 3.1]	-3.1 [-9.6, 3.2]	n.s
Diastolic BP, mmHg	Exam 1	76.2 ± 8.9	82.0 ± 8.3	88.6 ± 11.3	< 0.01
	Exam 2	71.2 ± 8.1	77.3 ± 8.8	82.3 ± 9.0	< 0.01
	Exam 3	70.1 ± 7.2	77.4 ± 9.5	80.3 ± 11.0	< 0.01
	Δ%	-6.1 [-13.5, -0.6]	-6.5 [-13.1, 2.6]	-8.0 [-16.9, 0.9]	n.s
Total Cholesterol, mg/dL	Exam 1	194.0 ± 29.0	244.6 ± 35.6	232.3 ± 35.4	< 0.01
	Exam 2	190.2 ± 28.3	237.6 ± 30.0	212.3 ± 34.1	< 0.01
	Exam 3	199.6 ± 28.4	241.0 ± 32.6	209.0 ± 35.0	< 0.01
	Δ%	3.8 [-4.3, 12.8]	0.8 [-9.0, 8.6]	-9.9 [-16.8, -0.4]	< 0.01
LDL, mg/dL	Exam 1	104.6 ± 26.3	155.1 ± 35.8	141.6 ± 33.3	< 0.01
	Exam 2	113.7 ± 23.4	158.7 ± 25.5	137.4 ± 31.5	< 0.01
	Exam 3	120.5 ± 22.7	161.8 ± 28.2	134.5 ± 32.3	< 0001
	Δ%	17.1 [2.0, 35.2]	7.6 [-5.4, 22.8]	-3.1 [-12.4, 9.1]	< 0.01
HDL, mg/dL	Exam 1	62.1 ± 18.7	57.3 ± 15.2	45.8 ± 12.8	< 0.01
	Exam 2	58.4 ± 16.4	54.4 ± 12.1	45.8 ± 10.4	< 0.01
	Exam 3	69.5 ± 20.2	61.2 ± 14.4	51.4 ± 14.9	< 0.01
	Δ%	14.6 [0.7, 26.0]	8.4 [-6.9, 23.3]	11.0 [-2.0, 22.3]	n.s
BMI, kg/m^2	Exam 1	23.9 ± 2.8	26.2 ± 2.3	30.8 ± 3.2	< 0.01
	Exam 2	24.4 ± 2.9	26.9 ± 2.8	32.0 ± 3.6	< 0.01
	Exam 3	24.9 ± 3.3	27.6 ± 3.2	32.9 ± 4.3	< 0.01
	Δ%	3.2 [-2.4, 8.8]	4.3 [0.7, 8.8]	7.1 [0.2, 13.5]	0.04
Waist Circumference, cm	Exam 1	81.8 ± 9.5	90.1 ± 7.7	102.7 ± 7.4	< 0.01
	Exam 2	83.8 ± 10.1	93.0 ± 7.2	108.3 ± 9.2	< 0.01
	Exam 3	87.9 ± 10.7	98.0 ± 8.5	113.3 ± 10.1	< 0.01
	Δ%	6.3 [1.1, 12.0]	7.9 [3.7, 13.1]	10.2 [4.8, 16.3]	0.03
HbA1c, %	Exam 1	5.4 ± 0.4	5.4 ± 0.3	5.6 ± 0.7	< 0.01
	Exam 2	5.3 ± 0.3	5.4 ± 0.3	5.8 ± 0.8	< 0.01
	Exam 3	5.3 ± 0.4	5.5 ± 0.4	6.1 ± 1.2	< 0.01
	Δ%	-1.1 [-6.0, 4.0]	1.4 [-4.4, 6.2]	5.9 [-2.4, 12.4]	< 0.01
LDL to HDL ratio	Exam 1	1.9 ± 0.8	2.9 ± 1.1	3.4 ± 1.3	< 0.01
	Exam 2	2.1 ± 0.8	3.1 ± 0.9	3.1 ± 0.9	< 0.01
	Exam 3	1.9 ± 0.7	2.8 ± 1.0	2.8 ± 1.1	< 0.01
Total Cholesterol to HDL ratio	Exam 1	3.4 ± 1.0	4.6 ± 1.4	5.5 ± 1.8	< 0.01
	Exam 2	3.5 ± 0.9	4.6 ± 1.1	4.8 ± 1.2	< 0.01

	Exam 3	3.1 ± 0.8	4.2 ± 1.2	4.4 ± 1.4	< 0.01
Lipid-lowering Medication	Exam 1	1 (0.9%)	2 (1.6%)	2 (2.4%)	n.s
	Exam 2	2 (1.8%)	9 (7.0%)	9 (11.0%)	0.02
	Exam 3	3 (2.6%)	14 (10.9%)	15 (18.3%)	<0.01
Antihypertensive medication	Exam 1	1 (0.9%)	9 (7.0%)	15 (18.3%)	<0.01
	Exam 2	6 (5.3%)	15 (11.6%)	22 (26.8%)	<0.01
	Exam 3	13 (11.4%)	29 (22.5%)	35 (42.7%)	<0.01
Validated Glycemic Status					<0.01
Normoglycemic	Exam 3	102 (89.5%)	81 (62.8%)	22 (26.8%)	
Prediabetes	Exam 3	10 (8.8%)	37 (28.7%)	30 (36.6%)	
Diabetes	Exam 3	2 (1.8%)	11 (8.5%)	30 (36.6%)	

Risk factor values at each time point are presented as arithmetic mean with standard deviation. Δ% is calculated as (value_[Exam 3]–value_[Exam 1])/value_[Exam 1]*100 and is presented as median with 1st and 3rd quartile. P-values from t-test or Wilcoxon rank test, where appropriate. Additional information for lipid-lowering medication, antihypertensive medication and validated glycemic status at Exam 3 is given as counts and percentages.

Supplementary Table S3: Goodness of Fit as measured by explained variance (adjusted R^2) of different models.

outcome	Predictor variables in model					
	**risk profile Exam 1 only**	**risk profile Exam 2 only**	**risk profile Exam 3 only**	**traj clusters only**	**traj clusters + risk profile Exam 1**	**traj clusters + risk profile Exam 3**
**TAT**	0.57855	0.74993	0.88847	0.54222	0.65176	0.88946
**VAT**	0.61280	0.69311	0.75721	0.62927	0.66006	0.76312
**SAT**	0.57030	0.73082	0.86844	0.48148	0.63180	0.86804
**RSFF**	0.28550	0.31595	0.32937	0.28467	0.28631	0.33040
**HFF**	0.43344	0.50097	0.53093	0.43124	0.47394	0.53285
**PFF**	0.21882	0.25570	0.25369	0.24432	0.25740	0.27140

All models are adjusted for age, sex, antihypertensive medication, lipid-lowering medication, smoking and validated diabetes. Note that the values in the first three columns are graphically displayed in Figure 1 in the main document. TAT: Total adipose tissue, VAT: Visceral adipose tissue, SAT: Subcutaneous adipose tissue, RSFF: Renal sinus fat fraction, HFF: Hepatic fat fraction, PFF: Pancreatic fat fraction

Supplementary Figure S1: Goodness-of-Fit of the linear regression models estimating the association of single-point risk profiles with adipose tissue outcomes. On the x-axis: single time points at which risk profiles were obtained: Exam 1, Exam 2, Exam 3. On the y-axis: Goodness-of-Fit as measured by explained variance in outcome (adjusted R2). The single time points are connected by lines for visual aid only. A): The risk factor profiles included systolic blood pressure, diastolic blood pressure, BMI, WC, Total Cholesterol, HDL, LDL and HbA1c whereas the outcome variables comprised TAT, SAT, VAT, RSFF, log (HFF) and log (PFF). B): The risk factor profiles included systolic blood pressure, diastolic blood pressure, Total Cholesterol, HDL, LDL and HbA1c whereas the outcome variables comprised BMI, WC, TAT, SAT, VAT, RSFF, log (HFF) and log (PFF)

TAT: Total adipose tissue, VAT: Visceral adipose tissue, SAT: Subcutaneous adipose tissue, RSFF: Renal sinus fat fraction, HFF: Hepatic fat fraction, PFF: Pancreatic fat fraction

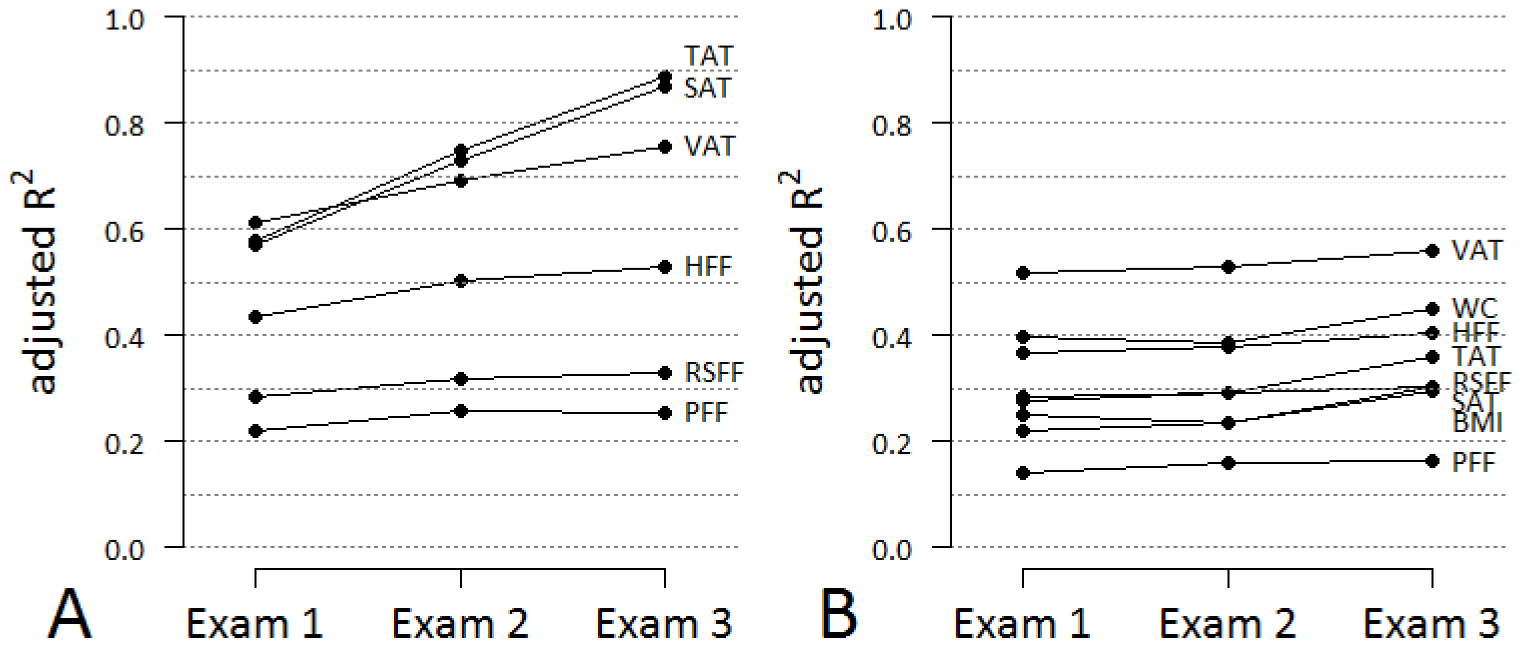

Supplementary Figure S2: Box plots illustrating the distribution of adipose tissue depots, measured at Exam 3, according to cluster membership of participants, when BMI and WC are excluded from the risk factor set and instead included as outcome variables. Cluster membership in either Cluster I, Cluster II or Cluster III was determined by multivariate k-means clustering based on individual longitudinal risk profile trajectories.

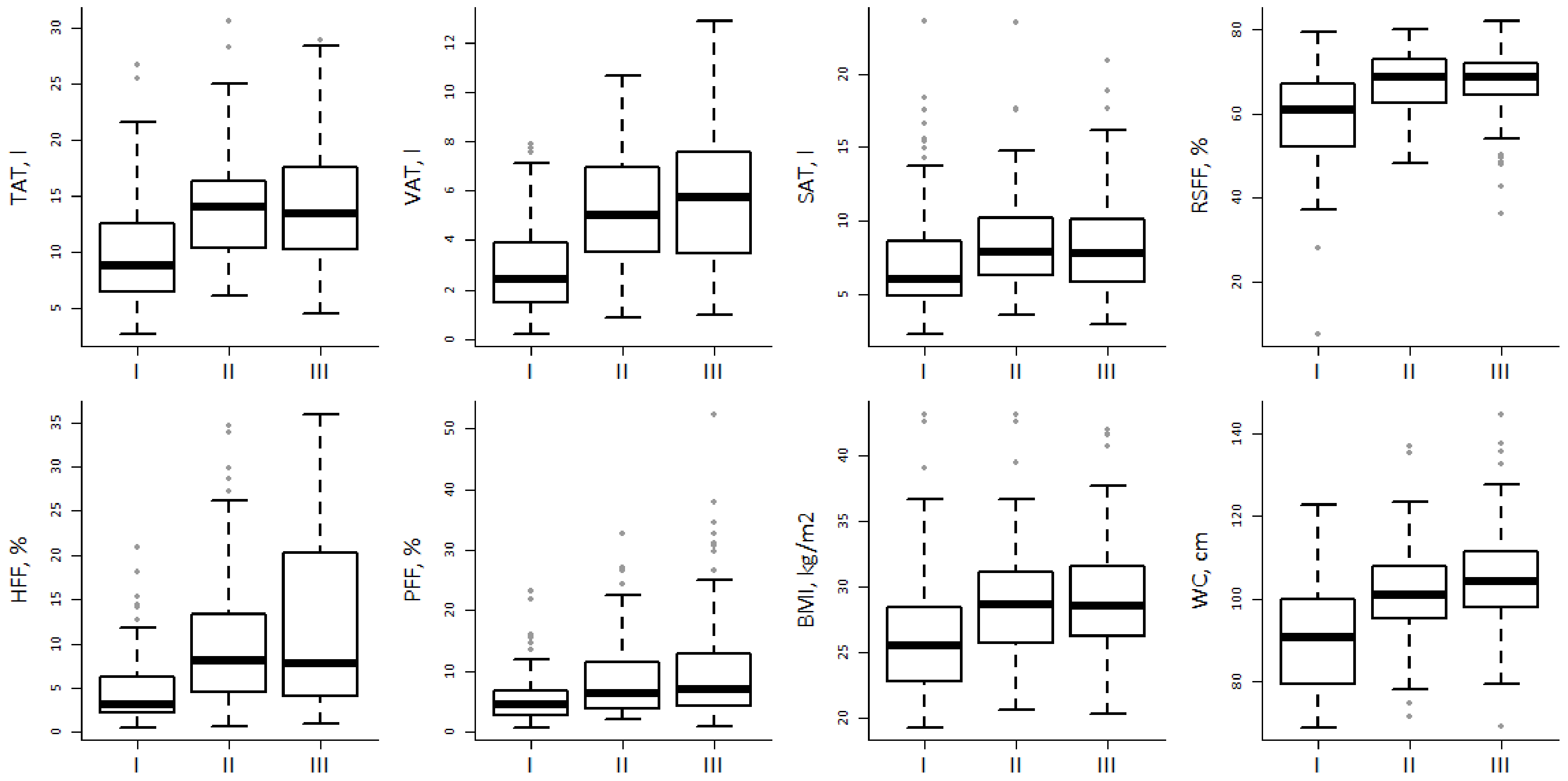

Supplement: Supplementary file 1 — Supplementary Material to Association of longitudinal risk profile trajectory clusters with adipose tissue depots measured by magnetic resonance imaging [file 41598_2019_53546_MOESM1_ESM.pdf]
